# Genetic characterization of a reptilian calicivirus (Cro1)

**DOI:** 10.1186/1743-422X-9-297

**Published:** 2012-11-29

**Authors:** Carlos Sandoval-Jaime, Gabriel I Parra, Alvin W Smith, Kim Y Green, Stanislav V Sosnovtsev

**Affiliations:** 1 Caliciviruses Section/LID/NIAID/NIH, Bethesda, MD 20892, USA; 2 Laboratory for Calicivirus Studies, Oregon State University, Corvallis, OR, 97330, USA

**Keywords:** Reptile calicivirus, Cro1, Complete genome, Vesivirus phylogeny

## Abstract

**Background:**

Vesiviruses in the family *Caliciviridae* infect a broad range of animal hosts including mammals, birds, fish, amphibians and reptiles. The vesivirus Cro1 strains were isolated from diseased snakes in the San Diego zoo in 1978 and reported as the first caliciviruses found in reptiles. The goal of this study was to characterize the Cro1 strain 780032I that was isolated in cell culture from a rock rattlesnake (*Crotalus lepidus)* in the original outbreak.

**Results:**

We re-amplified the original virus stock in Vero cells, and determined its full-length genome sequence. The Cro1 genome is 8296 nucleotides (nt) in length and has a typical vesivirus organization, with three open reading frames (ORF), ORF1 (5643 nt), ORF2 (2121 nt), and ORF3 (348 nt) encoding a nonstructural polyprotein, the major capsid protein precursor, and a minor structural protein, respectively. Phylogenetic analysis of the full-length genome sequence revealed that the Cro1 virus clustered most closely with the VESV species of the genus *Vesivirus*, but was genetically distinct (82-83% identities with closest strains).

**Conclusions:**

This is the first description of a full-length genome sequence from a reptile calicivirus (Cro1). The availability of the Cro1 genome sequence should facilitate investigation of the molecular mechanisms involved in Cro1 virus evolution and host range.

## Background

The family *Caliciviridae* is a large group of small, non-enveloped RNA viruses that includes important human and animal pathogens [1]. The family is comprised of five genera: *Lagovirus, Vesivirus, Nebovirus, Sapovirus* and *Norovirus*[2] and two new genera have been proposed [3]. Despite marked genetic and antigenic diversity, caliciviruses share several common features. All have icosahedral virions with a protein shell containing 180 copies of a major capsid protein, VP1 [4]. The virions carry a positive-sense single-stranded RNA genome of approximately 6.4 to 8.5 kb in length. The 3’-end of the calicivirus RNA is polyadenylated, and the 5’-end is covalently linked to a small protein encoded by the virus genome, VPg [5]. Calicivirus RNA genomes share similar organization; they are comprised either of two or three ORFs. The large ORF1 encodes the virus nonstructural proteins and is expressed from the genomic RNA template. In the genomes of sapo-, lago-, and neboviruses, the nonstructural region is fused to the gene encoding virus capsid protein, VP1. In the genomes of vesi- and noroviruses, the capsid protein is encoded by a separate ORF2, located towards the 3’-end of the virus genome. For all caliciviruses, the capsid proteins are produced from an abundant subgenomic RNA synthesized during virus replication. The same RNA serves as bicistronic template for the expression of a minor capsid protein, VP2. The ORF encoding VP2 is near the 3’-end of the virus genome and is conserved among caliciviruses [5].

The genus *Vesivirus* currently contains two approved species, Vesicular exanthema of swine virus (VESV) and Feline calicivirus (FCV), and a diverse group of unassigned, phylogenetically-related viruses [6]. There are several well-recognized animal pathogens among vesiviruses that have been associated with a variety of disease conditions. These include diarrheal disease in dogs [7], respiratory illness, vesicular lesions, and epidemic hemorrhagic fever in cats [8,9], and vesicular lesions in several other host species including swine, pinnipeds and humans [10-12]. The prototype virus of the genus *Vesivirus*, VESV, was originally isolated from pigs with clinical signs compatible with those caused by infection with foot-and-mouth disease virus (FMDV) [11]. In 1972, a virus with morphological and biochemical characteristics indistinguishable from those of VESV was isolated on San Miguel Island, California from sea lions and named San Miguel Sea lion virus (SMSV) [13,14]. When the experimental infection of pigs with SMSV resulted in a vesicular disease clinically mimicking FMDV and VESV infection, marine animals including ocean fish were retrospectively implicated to be the original source of VESV outbreaks [13,14]. Since then, VESV, SMSV, and other related caliciviruses have frequently been designated as the “marine vesiviruses.” In contrast to FCV, which are considered to have restricted host specificity to cats of the family *Felidae*, the marine vesiviruses have been described as having an unusually broad host range [15-18].

In 1978–79, sixteen new vesivirus strains were isolated from four poikilothermic species (Aruba Island rattlesnake, *Crotalus unicolor,* Rock rattlesnake*, Crotalus lepidus,* Eyelash viper *Bothrops schlegeli,* Bell’s horned frog *Ceretophyrs ornate)* in a California zoological collection. The sixteen viruses were antigenically related and were not neutralized by the available VESV-like reference sera [19]. The new viruses were proposed as members of a new reptilian caliciviruses (RCV) Crotalus 1 (Cro1) serotype [19]. Sequence analysis of a 453 nt region of the Cro1 polymerase gene provided additional evidence for a new vesivirus group [20]. The Cro1 serotype did not appear to be restricted geographically or temporally, or limited to reptile and amphibian hosts. In 1986–7, vesiviruses neutralized by the Cro1 typing serum were isolated from samples collected from three different marine mammals species (*Eumetopias jubatus, Zalophus californianus californianus,* and *Callorhinus ursinus*) along the coast of Oregon and California states [16].

In this study, we determined the full-length genome sequence of Cro1 strain 780032I, isolated from the intestine of a Rock rattlesnake (*Crotalus lepidus*) housed in the San Diego Zoo in 1978. Comparison of the genome sequence of 780032I with those available in GenBank database shows that this Cro1 virus represents a genetically distinct vesivirus strain within the species VESV.

## Results and discussion

**Figure 1:**
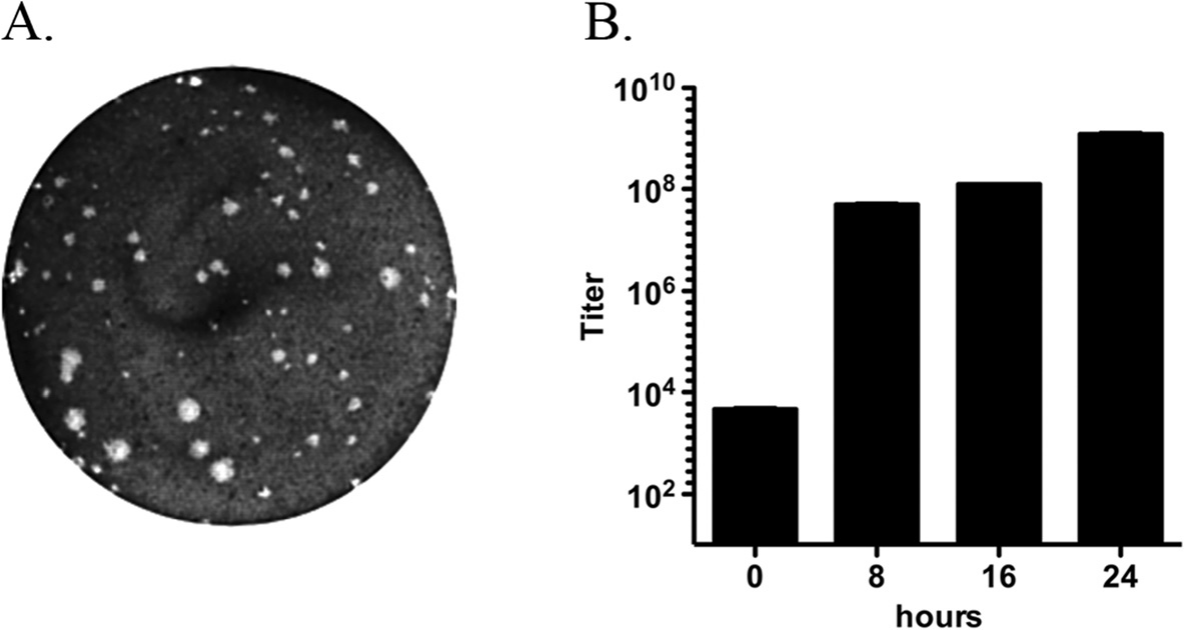
***In vitro *growth characterization of the 780032I strain isolated from *Crotalus lepidus*.A**) The growth of the 780032I strain results in lysis of infected Vero cells and in the subsequent formation of plaques in a cell monolayer overlayed with 1% agarose containing growth medium. Plaques can be visualized with crystal violet staining after cells are fixed with formaldehyde. **B**) The virus titers of the 780032I strain produced by the Vero cells. The cell monolayers were infected with MOI=0.01 and virus titer was measured at different time points by plaque assay. The titers shown are expressed as the mean from two independent experiments performed in duplicate.

Cro1 virus stocks obtained from snake samples collected during the original outbreak in the San Diego Zoo were examined for their ability to grow in cell culture following decades of storage at −80°C. In a preliminary screening, virus sample #780032I obtained from a Rock rattlesnake (*Crotalus lepidus*) showed efficient growth in Vero cells (CCL-81, ATCC, Manassas, VA). Strong cytopathic effect (CPE) was observed in the virus infected cell monolayer at 24–48 hours post infection (hpi). Plaque titration with an agarose overlay resulted in the formation of detectable plaques at 48 hpi (Figure 1A). The efficiency of virus replication was examined in a multiple-cycle growth curve time course analysis. Inoculation of Vero cells at multiplicity of infection (MOI)=0.01 consistently produced titers of ~10^8^-10^9^ pfu/ml by 24 hours (Figure 1B). To verify virus identity, four individual virus plaques were collected in agarose plugs and virus RNA was extracted from each of them using the RNeasy Kit (Qiagen, Valencia, CA). Purified RNA was employed for the RT-PCR amplification of the virus subgenomic region followed by direct sequencing of the ORF2. Comparison of the ORF2 nucleotide sequences with those in GenBank (AY772540 and AY772541) confirmed the identity of the 780032I strain as Cro1. No evidence of genetic variation was observed among plaque-purified viruses.

**Figure 2:**
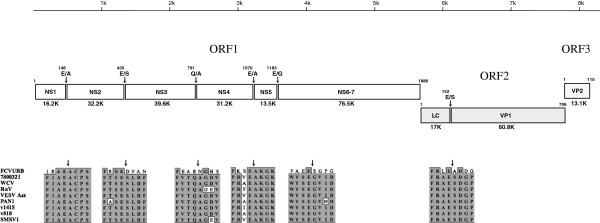
**Schematic representation of the 780032I strain genome organization.** The 780032I genome is comprised of three major ORFs. ORF1 encodes a nonstructural polyprotein, ORF2 – a precursor of the virus capsid protein, VP1, and ORF3 – a minor capsid protein, VP2. Mature virus proteins are shown as rectangular boxes with calculated protein molecular masses indicated below. The putative borders of the virus mature nonstructural proteins are indicated with predicted cleavage sites and arrows. The E^152^/S site that is likely cleaved by the virus-encoded protease during capsid protein maturation is shown for the ORF2-encoded precursor. The 780032I predicted cleavage sites in the ORF1 were based on an alignment of VESV-like ORF1 polyprotein sequences with the established map of FCV [23].

Re-amplified 780032I stock was employed for virus RNA isolation, cDNA amplification and direct sequencing analysis. To determine the full-length genome sequence, several overlapping cDNA fragments of the virus genome were RT-PCR amplified, and sequenced on both strands using a primer-walking technique. The complete genome sequence of the 780032I strain contained 8296 nt, excluding the poly (A) tail. Like other vesiviruses, it was comprised of three predicted open reading frames (ORF1, 2, 3) bordered by short 5’- and 3’-end nontranslated (NTR) sequences (Figure 2). The 5’-end NTR sequence of the 780032I-strain genome was 19 nt in length and was highly conserved among VESV-related vesiviruses (VESV A48, PAN1, RaV, WCV, SMSV1, v810 and v1415). The 3’-end NTR was 164 nt in length, and showed a higher degree of nucleotide variation when compared to that of other vesiviruses. The lengths of the ORF1, ORF2 and ORF3 were found to be 5643 nt, 2121 nt and 348 nt, respectively. The stop codon of ORF1 (in bold) and the start codon of ORF2 (underlined) were separated by five nucleotides (**UAG**CCAUUAUG), while ORF2 and ORF3 overlapped by four nucleotides (A**UGA**) similar to other vesiviruses. The 3’-end of the 780032I ORF1 (nt 5648–5660) contained a conserved sequence motif that showed a high level of identity (10 out of 13 nt) with the first 13 nt of the virus genome. In addition, this motif was highly similar (9 out of 13 nt) to the 5’-end of the FCV subgenomic RNA that was mapped by primer extension analysis [21,22]. Taken together, these data supported the predicted start of the 780032I subgenomic RNA as position 5648 of the genome.

The vesivirus ORF1 encodes a nonstructural polyprotein that undergoes co-translational proteolytic processing during virus replication [23]. A cascade of proteolytic events mediated by the virus-encoded proteinase gives rise to the virus mature nonstructural proteins and their intermediate forms. The Cro1 ORF1 encodes an 1880 amino acid nonstructural polyprotein with a predicted molecular mass of 209 kDa. Motif scanning [24,25] and comparative sequence analysis of the Cro1 polyprotein revealed the presence of the characteristic NTPase (^590^GppgcGKT^597^), 3C-like proteinase (^1303^GDCG^1306^) and 3D-like RNA-dependent RNA polymerase (^1653^GLPSG^1657^ and ^1701^YGDD^1704^) catalytic motifs in an order conserved among caliciviruses. Alignment of the Cro1 ORF1 polyprotein sequence with that of another vesivirus, FCV, that had an experimentally established cleavage map [23] allowed prediction of the putative cleavage sites and, correspondingly, the sizes of mature nonstructural proteins. The predicted Cro1 polyprotein cleavage sites were conserved among VESV-related vesiviruses and, with the exception of cleavage site between NS3 and NS4 proteins, all carried a glutamic residue in the P1 position and alanine, serine or glycine residues at the position P1’ (Figure 2). Of interest, the NS3-NS4 scissile bond was predicted between glutamine and alanine residues. The order and sizes of the proteins defined by these cleavage sites were as follows: 16.2 kDa NS1 – 32.2 kDa NS2 – 39.6 kDa NS3^NTPase^ – 31.2 kDa NS4 – 13.5 kDa NS5^VPg^ – 76.5 kDa NS6-7^ProPol^.

The vesivirus ORF2 encodes a precursor of the virus capsid protein that is processed by cleavage during capsid protein maturation. Processing of the FCV capsid precursor at an E^124^-A^125^ dipeptide by virus NS6-7^ProPol^ results in removal of an 124 aa-long N-terminal leader sequence (LC) and release of the 59–60 kDa mature form of the virus capsid protein, VP1 [26]. The Cro1 ORF2 sequence is predicted to encode a precursor protein with a molecular mass of ~78 kDa. Alignment with the FCV capsid precursor sequence showed the presence of a putative scissile bond between ^152^E and ^153^S (Figure 2). It is likely that, similar to FCV, this cleavage site defines the border between the virus capsid protein leader sequence and mature VP1. Of interest, the site is conserved in the corresponding sequences of the recently characterized v810 and v1415 vesivirus strains isolated from Steller sea lions [27]. The expression of the v810 and v1415 ORF2 sequences that were N-terminally truncated at this site resulted in production and self-assembly of virus-like particles morphologically and antigenically similar to virions [28].

The small ORF3 located at the 3’-end of the vesivirus genome encodes a minor component of virus capsids, the VP2 protein [29]. The protein is expressed from the virus bicistronic subgenomic RNA and has been shown to be essential for the formation of infectious vesivirus virions [30]. The substitution of a C for a T at nucleotide 8115 converted a stop codon (TAG) to a glutamine codon (CAG) resulting in a five codon-extension of the ORF3 compared to other VESV-related vesiviruses (data not shown). The 3’-end of the calicivirus RNA genome has been shown to play a crucial role in the initiation of virus replication [31,32]. Nevertheless, modifications introduced into the genomes of feline calicivirus and murine norovirus showed that this region could tolerate a number of sequence changes while supporting virus replication [30,32].

To elucidate the genetic and phylogenetic relationships of the Cro1 virus with other vesiviruses, multiple sequence alignments of the representative vesivirus genome sequences were generated using the ClustalW algorithm in the Mega5 software package [33]. Comparative genetic distance analysis of the vesivirus sequences revealed the presence of three genetically distinct virus groups, provisionally called here as VESV-like (or marine vesiviruses), FCV-like and CaCV-like viruses. The newly characterized Cro1 virus clustered with the VESV-related group, where differences in sequence identity did not exceed 23%. Pairwise comparison of the Cro1 genome sequence with those of VESV-like viruses showed that the genome of the most distant VESV-like virus, walrus calicivirus, differed from that of Cro1 by 21%. The genetic distances observed between genomes of Cro1 and FCV-like, and CaCV-like viruses were significantly higher at 48% and 50%, respectively (Table 1). Of interest, FCV and CaCV were most distant within the genus, with differences in genome sequences reaching 56% (data not shown).

**Table 1:** Percent nucleotide and amino acid identity of Cro1 strain 780032I with other versiviruses

**780032I**	**genome**	**NS1**		**NS2**		**NS3**		**NS4**		**NS5**		**NS6-7**		**LC**		**VP1**		**VP2**	
		**nt**	**aa**	**nt**	**aa**	**nt**	**aa**	**nt**	**aa**	**nt**	**aa**	**nt**	**aa**	**nt**	**aa**	**nt**	**aa**	**nt**	**aa**
VESV A48	82.9	91	87.8/92.6	83.7	90.6/96.9	80.2	91.3/96.6	88.5	95/97.1	78.5	80.5/90.3	86.2	91.7/96	82.7	81.6/88.2	74.6	80.4/87.1	80.2	86.1/93
PAN1	82.5	89.2	87.2/91.2	82.1	89.5/96.5	79.9	91.9/96.9	88.1	93.6/96.4	85	95.6/98.2	83.9	90.8/95.6	85.6	81.6/90.1	75.2	82.6/89.6	81.6	85.2/89.6
v1415	82.1	84.2	79.7/83.1	80.3	89.2/95.8	80.8	93.8/98	88.3	93.5/96.8	82.3	93.8/99.1	82.7	93.5/97.6	84.2	89.5/94.7	77	84.9/90.5	81.3	85.2/90.4
v810	82.5	86	80.4/87.8	78.4	88.5/94.8	80	92.4/97.2	87	94.6/97.5	83.2	95.6/100	82.8	93.1/97.7	87.1	89.5/94.1	78.9	85.3/91	84.8	89.6/92.2
SMSV1	79.2	88.3	85.8/89.2	81.6	87.5/95.1	80.1	90.4/96.9	89.5	94.3/97.1	83.5	94.7/100	84.4	91.5/96.4	80.7	83.6/89.5	64.5	67.2/80.4	68.4	68.7/80.9
WCV	78.9	89	85.1/90.5	80.6	88.9/95.8	79.8	92.1/97.5	87	92.1/96.1	84.1	93.8/100	85.9	92.4/97	81.4	82.9/90.8	63.9	66/80.1	67	73.9/83.5
RaV	79.2	92.1	88.5/90.5	82	89.5/95.8	80.4	91.3/97.2	86.4	92.5/95.3	82.9	90.3/98.2	86	93.1/97.3	78.3	81.6/86.8	63.6	66.1/79.9	65.5	71.3/81.7
CaCV	49.7	29.7	7.4/22.2	54.1	46.9/65.4	57.9	57.9/75	55.2	51.9/66.6	49.3	40.7/65.9	58.3	56.9/71.1	41.6	30.6/43.1	46.1	38.6/52.7	32.9	29.4/40.4
GCV8	51.2	38.1	6.1/19.6	53.1	46.2/65.1	59.6	57.3/75.8	55.9	52.6/67.7	50.9	39.8/63.4	60.3	60.3/74.7	45.5	27.5/40	46.2	38/54	31.4	26.5/38.2
ACV8	50.7	38.3	6.7/20.1	53.8	46.9/65.4	59.5	57.6/75.8	56.4	51.9/67.7	52	39.8/63.4	59	60.3/74.6	44.1	26.9/39.4	45.8	38.5/54.9	31	26.5/38.2
ACV9	50.5	38.5	6.7/20.1	53.1	46.6/65.4	59.6	57.6/75.8	56.2	52.3/67.7	52	39.8/63.4	59	60.3/74.6	43.7	26.9/39.4	45.8	38.5/54.7	30.7	26.5/38.2
FCVURB	52.2	12.8	4.1/8.8	55.2	48.6/68.1	62.2	63.2/79.8	55.6	51.1/70	57.3	58.8/71.1	58.2	59.1/74.9	32.5	19/30.7	53.7	50.6/67.9	41.7	20/37.4
FCVF65	51.6	12.6	4.7/8.8	54.2	45.8/65.3	61.6	59.8/76.7	56.1	50/68.9	56.1	56.1/68.4	57.6	58.2/73.5	30.8	19/30.1	52.9	49.6/66.8	40.9	20/37.4
FCVF4	52.6	13.2	4.1/8.8	53.6	47.2/67.4	63.9	63.8/79.5	56.4	51.1/69.6	56.7	57.9/69.3	58.8	58.7/74.6	31.9	19.6/30.7	54.3	51.2/67	40.9	20.9/38.3
FCVF9	51.6	13	4.1/8.8	53	47.2/66.7	61.5	64.3/79.5	56.2	51.8/70.4	54.4	56.1/71.1	57.9	58.8/74.9	33	18.3/30.1	53.7	51.2/67.7	39.4	20/36.5

Relatedness of amino acid sequences are shown as percent identity/percent similarity.

**Figure 3:**
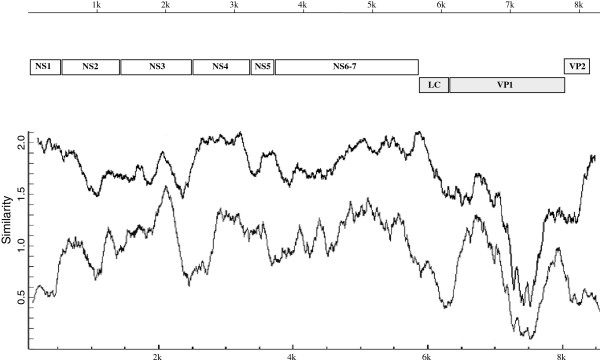
**Schematic representation of the vesivirus genome organization and the sequence conservation along the alignment of the full-length genome sequences.** The Plotcon program was used for the sequence comparison of either the VESV-like viruses only (black color line) or all vesiviruses (grey color line). Similarity plots were generated with a sliding window of 200 nt. Each plotted point was an average of the position similarities within the window, and the position similarities were calculated using the algorithm described in plotcon ( http://emboss.open-bio.org/rel/rel6/apps/plotcon.html).

The genome variability of viruses within the VESV-like group was analyzed using the Plotcon algorithm in the EMBOSS software package [34]. A similarity profile generated for the multiple sequence alignment of VESV-like viruses revealed the presence of conserved and variable regions across the virus genome (Figure 3). The relatively well conserved sequences included virus nonstructural NS1 and NS4 genes, and the region corresponding to the ORF1-ORF2 junction known to contain a putative transcription start of the virus subgenomic RNA. The VESV-like NS1 and NS4 genes shared an increased level of average nucleotide sequence identity (88.6%) compared to that of the entire ORF1 (84.6%). As expected, the most variable part of the virus genome was observed in the ORF2 region that encodes the P domain of the virus capsid protein. The marked variation (35.4% nucleotide difference) was consistent with structural and functional studies that have shown that this region of the VP1 is exposed on the surface of the virion and plays a role in receptor binding and immune selection in the host [35]. Of interest, similarity plots for the deduced amino acid sequences encoded by ORF1, 2 and 3, had profiles similar to those of the corresponding nucleotide regions of the virus genome (data not shown).

The profiles of the genome plots generated in a comparison of VESV-, FCV- and CaCV-like vesivirus sequences as well as that for VESV-like sequences alone were similar. Consistent with the generally higher level of genetic divergence existing between vesiviruses from different groups, the similarity indexes calculated for the all-vesivirus sequence alignments were significantly lower. Interestingly, the comparative analysis showed that the NS1 and part of the NS4 sequence were not conserved when compared to the corresponding sequences of the FCV-like and CaCV-like viruses (Figure 3). Furthermore, predicted vesivirus NS1 gene sequences varied in length from 138 to 534 nt between these groups of viruses. For the Cro1, the nucleotide identity of the NS1 gene (predicted to be 148 aa long) was 29.7-38.5% with the corresponding sequences of CaCV-like viruses, and 51.6-52.2% with those of FCV-like viruses. The level of similarity for the NS1 deduced amino acid sequences was calculated to be 19.6-22.2% and 8.8% between Cro1 and CaCV-like and between Cro1 and FCV-like viruses, respectively. The function of the calicivirus NS1 protein is unknown and cannot be predicted based on the protein sequence since it does not show significant homology with any established functional sequence motifs. Moreover, the level of similarity of NS1 amino acid sequences between different calicivirus genera does not exceed that of random sequences, precluding identification of conserved sequence motifs.

The observed level of the NS4 sequence identity was significantly lower when the Cro1 sequence was aligned to those of FCV- and CaCV-like viruses. For the latter two, it ranged from 55.2 (Cro1 vs FCV) to 56.4% (Cro1 vs CaCV). In contrast, the lowest level of sequence identity among VESV-like virus NS4 genes was 86.4%. The similarity profile showed that the most variable sequences of the NS4 gene were located near the 5’-end (Figure 3). Similar to NS1, the NS4 gene is not conserved among viruses from different *Caliciviridae* genera and represents the second most variable region in the nonstructural ORF of their genomes. Nevertheless, all calicivirus NS4 proteins share a conserved structural feature, which is the presence of a hydrophobic domain. Of interest, a cluster of hydrophobic amino acid residues is located near the C-terminus of the Cro1 NS4 (MacVector’s Protein Analysis Toolbox), with amino acids 249–271 predicted to form a membrane-associated helix (TMpred server). Consistent with the putative role of this domain in membrane interactions, biochemical studies showed that transiently expressed NS4 behaved as an integral membrane protein [36]. In addition, different forms of the NS4 were found to localize to the membrane-associated virus replication complexes in calicivirus infected cells [36,37]. The significant sequence diversity suggests that NS4 might play an important role in determining the specificity of protein-membrane interactions in the host cell. For example, the subcellular localization of the norovirus NS4 is determined by an ER export signal motif (MERES) conserved only among the noroviruses [38]. Moreover, the presence of the MERES motif was shown to be critical to the NS4 antagonist role in ER/Golgi trafficking [38,39]. Of interest, computational analysis showed that the MERES motif was not present in the Cro1 sequence. In addition, scanning of the Cro1 NS4 sequence with software designed to identify putative signal and subcellular localization motifs (see Materials and Methods) found no known targeting sequences. The presence of such signals in the Cro1 NS4 protein remains to be established.

Another region of marked sequence variation was observed downstream from the ORF1-ORF2 junction. Plotcon analysis of the vesivirus sequences revealed a low level of nucleotide identity among the virus LC genes, with the lowest identity (30.8%) between the Cro1 and FCV65 viruses. Accordingly, a lower level of similarity was found for the compared deduced amino acid sequences of this protein, 30.1-30.7% for FCV-Cro1 and 39.4-43.1% for CaCV-Cro1 pairs. The function of this protein remains unknown; however, cleavage of the LC from the capsid precursor molecule was crucial for production of infectious virus particles [26].

**Figure 4:**
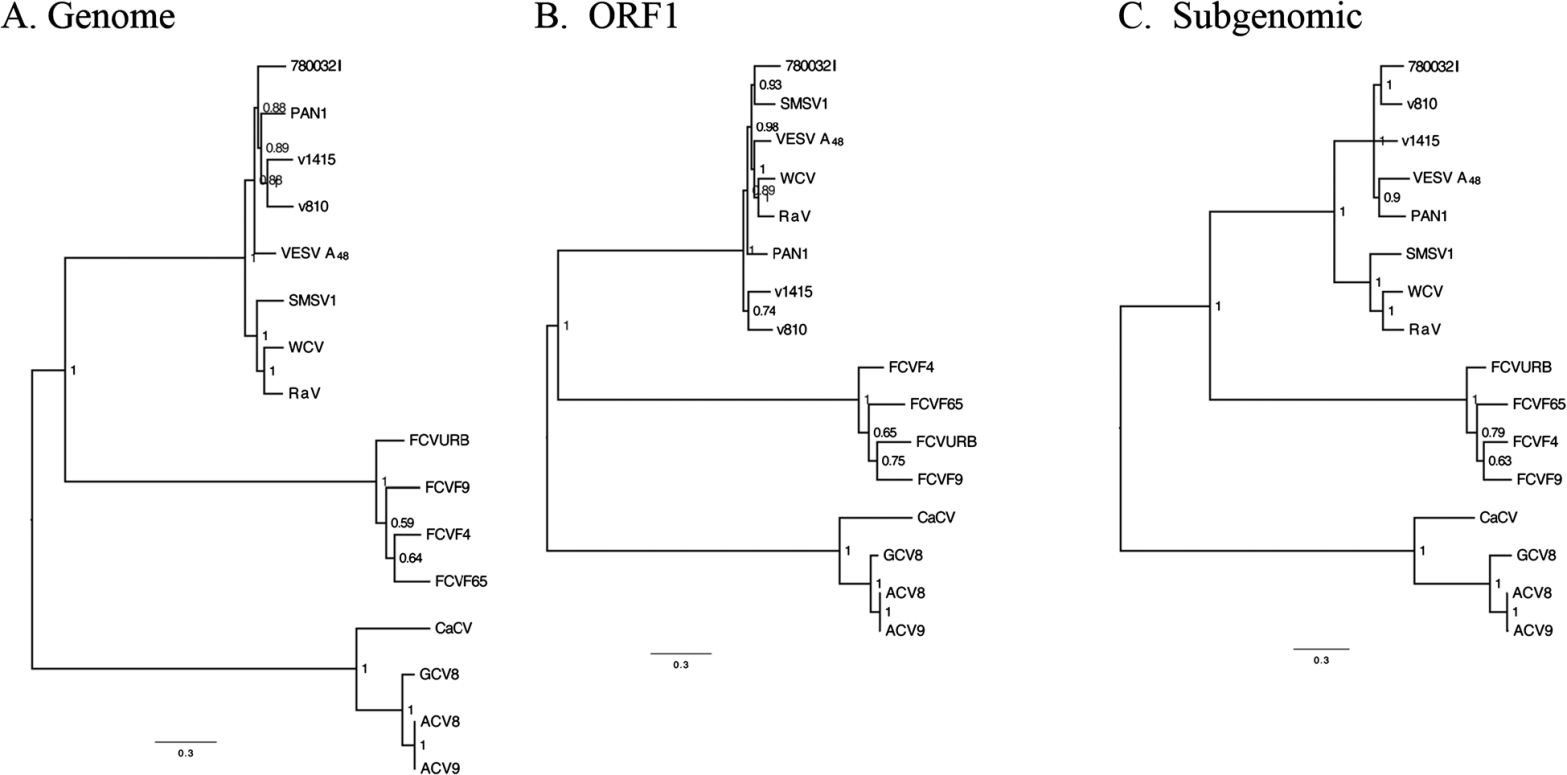
**Phylogenetic relationship of the 780032I strain isolated from reptile with other vesiviruses.** Phylogenetic trees for the alignments of the vesivirus full-length genome (**A**), ORF1 (**B**) and subgenomic RNA (**C**) sequences were inferred using Bayesian method (MrBayes 3.1.2) and parameters described in Materials and methods section. Bayesian clade probability values are shown next to the nodes.

To investigate the phylogenetic relationship of the Cro1 virus with other vesiviruses, a phylogenetic tree was inferred from multiple alignments of representative sequences using the Bayesian method. Figure 4A shows a consensus tree produced by MrBayes3.1.2 for the set of full-length genome sequences. A similar approach was employed to generate additional phylogenetic trees for the vesivirus ORF1 and subgenomic RNA sequences (Figure 4B and 4C). Analysis of the vesivirus genomic tree revealed the presence of three phylogenetic groups. The first group included FCV strains; the second group consisted of viruses related to the CaCV strain, and the third combined together viruses closely related to the genus prototype strain, VESV A48. A similar topology was observed for the phylogenetic trees inferred for the ORF1 and subgenomic RNA sequences. In all trees, the presence of three major clusters was strongly supported by a high posterior probability value for each clade.

Consistent with genetic distance analysis, the genomic sequence of the Cro1 780032I strain clustered together with those of the VESV-like viruses, making it a member of the marine vesivirus group (Figure 4). Within this cluster, the inferred phylogenetic relationships between RaV, SMSV1, VESV A48, PAN1, WCV and Steller sea lion v810 and v1415 strains were found to be similar to those reported earlier [17,27,40]. Of interest, when the phylogenetic tree was inferred for the virus subgenomic RNA sequences, the 780032I strain clustered together with the v810 strain. Similar clustering was observed when a set of the capsid sequences was extended to include two additional sequences available in GenBank, SMSV4 and SMSV17 (accession numbers M87482 and U52005, respectively). In the extended capsid tree, SMSV4 formed a separate group with VESV A48, while SMSV17 clustered with the 780032I, v810 and v1415 strains. However, the latter cluster showed a lack of strong statistical support, with the posterior probability value reaching only 0.68 (data not shown). Of note, the ORF1 and subgenomic sequences of the SMSV1 strain showed inconsistent grouping with those of RaV and WCV. The observed incongruence of the phylogenetic clustering of these viruses suggested a possible recombinant origin of the SMSV1 strain. Similarity plot analysis of the SMSV1 and other vesivirus genome sequences demonstrated an increased level of nucleotide sequence identity between the subgenomic regions of SMSV1 and RaV/WCV strains with a predicted putative recombination site near the junction of the SMSV1 ORF1 and ORF2 sequences (data not shown). However, our preliminary analysis could not identify the second parental strain or its probable lineage (data not shown). Confirmation of a possible recombination event will require further investigation.

**Figure 5:**
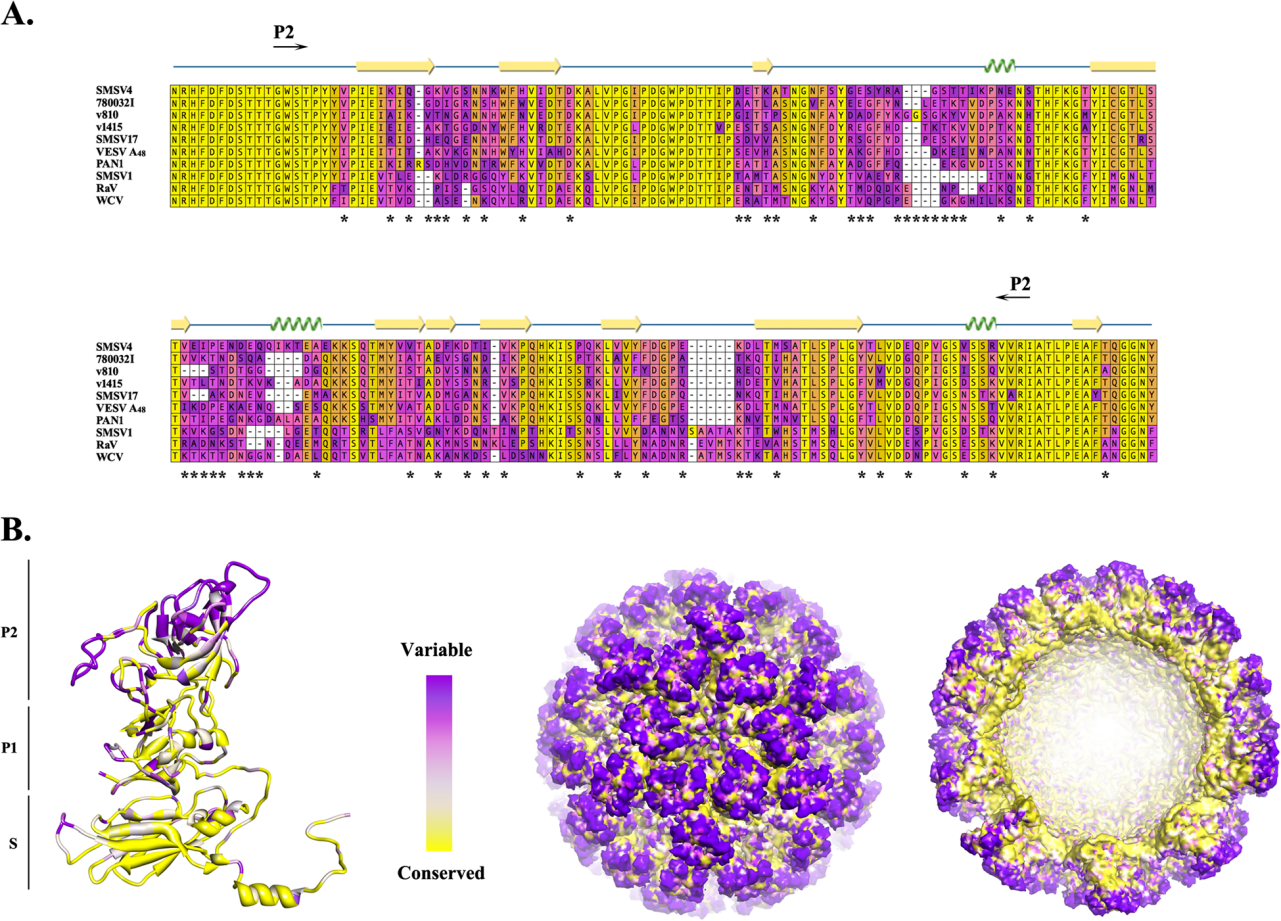
**Amino acid sequence variability of the VESV-like virus VP1 proteins.**. **A**) Multiple sequence alignment of the VP1 P2 regions of the VESV-like viruses Asterisks indicate positions where differences in amino acid sequences between strains v810 and 780032I are observed. Structural elements such as β−strands and α−helices found in the structure of SMSV4 VP1 are depicted above the alignment as hollow box arrows and coils, respectively. **B**) Ribbon and surface representation of the SMSV4 VP1 protein and its virus capsid assembly inferred using the Chimera program. Color coding of the alignment, ribbon structure and virus capsid were generated using the ConSurf software and is based on variability scale from 0 to 100%, with the most variable residues colored dark purple.

More than 40 serotypes of vesiviruses have been identified using serum-neutralization tests [41]. The virus capsid protein VP1 has been shown to encode major antigenic epitopes recognized by antibodies in polyclonal sera of infected animals. Correspondingly, the sequence variability of this protein is thought to provide the molecular basis for serotypic diversity of the vesiviruses. The highest similarity of amino acid sequence (91.4%) for the Cro1 VP1 protein was observed with the VP1 protein of a recently described v810 strain [28]. Interestingly, most (72 out of 79) of the amino acid changes in the VP1 sequence of these strains mapped to the C-terminal part of the protein, a region of the vesivirus genome with the highest level of variability (Figure 3). Evolutionary conservation scores generated from the corresponding multiple sequence alignment were mapped onto the structure of the SMSV4 VP1 protein with the aid of the ConSurf server software [42]. The X-ray structure of the SMSV4 VP1 (resolved to 3.2-Å) showed that the vesivirus capsid protein shared a domain organization similar to that of human noroviruses. The protein contained a short N-terminal arm (aa 10–48) and two domains, shell (S) and protruding (P), formed by its N-terminal (aa 49–209) and C-terminal (aa 210–554) parts, respectively. Based on the structure, the P domain could be further divided into two subdomains, P1 and P2, with the latter formed by the most distally located part of the protein [35]. The ConSurf analysis of the sequence variability coupled with visualization of the data in Chimera [43] showed that the most divergent parts of the P domain were localized on the surface of the virus capsid and were represented mainly by the sequence of the P2 subdomain (Figure 5A and 5B). It was noteworthy that 54 out of 72 amino acid differences observed between the VP1 P domains of the v810 and 780032I strains were mapped to the P2 region (Figure 5A). Structurally, the P2 sites that accumulated the majority of the sequence differences between these two viruses were located in five loops exposed on the surface of the virus capsid (Figure 5A and 5B). Surface localization of these loops and their flexibility are thought to play an important role in defining antigenic characteristics of the virus capsid and in its interactions with host cell receptors.

Our finding that Cro1 strain 780032I (isolated from Rock rattlesnake) shares strong sequence identity with the VESV-like marine vesiviruses is consistent with reports that the Cro1 serotype has become established in both aquatic and terrestrial hosts [16,19]. The original tissue samples from reptiles in the San Diego Zoo Cro1 outbreak of 1978 are no longer available for analysis, so it is impossible to investigate the outbreak retrospectively. Although the source of this Cro1 virus remains unknown, the continued genetic characterization of known viral isolates in concert with the increasing use of deep sequencing techniques for virus discovery may help track the origin and spread of this and other caliciviruses in nature.

## Materials and methods

### Sample isolation and virus amplification

Reptile tissue samples (small intestine, liver, kidney) were collected in 1978 from hatchling and breeding snakes in the San Diego Zoo. Snakes were experiencing high mortality rates thought to be associated with enteritis and hepatitis of an unknown cause [19]. The tissue samples were homogenized and clarified by low-speed centrifugation. To amplify the virus, the corresponding supernatants were filtered through 0.22 μm polysulfone filters, and the resulting filtrates were added to Vero cell monolayers maintained in Eagle’s minimal essential medium that was supplemented with penicillin (200 units/ml), streptomycin (100 μg/ml), L-glutamine (2 mM) and 10% heat-inactivated fetal bovine serum. The inoculated cells were monitored until the appearance of visible CPE. When CPE in monolayers exceeded 90%, virus stocks were generated by collection of growth media and low speed clarification of supernatants. After viruses were plaque-purified three times, the amplification procedure was repeated and the clarified supernatants were aliquoted and stored at −80°C.

### RNA extraction and RT-PCR sequencing

Viral RNA was extracted from virus stock samples with the RNeasy Mini Kit (Qiagen). The extracted RNA was used as a template for reverse transcription (RT) and PCR amplification of cDNA fragments. The RT-PCR reactions were performed using One-Step RT-PCR Kit (Invitrogen, Carlsbad, CA) and vesivirus genome-specific primer pairs. Briefly, following the initial RT reaction, 30 min at 45°C, a denaturation step at 94°C was performed for 2 min, followed by 40 cycles of 15 s at 94°C, 30 s at 50°C, and 3 min at 68°C. Amplified cDNA fragments were resolved by electrophoresis in 1.0% agarose gels containing ethidium bromide. The corresponding DNA bands were visualized with UV light and excised from the gel. The DNA was extracted with QIAquick Gel Extraction Kit (Qiagen) and subjected to nucleotide sequencing using the Big Dye Terminator v3.1 Cycle Sequencing Ready Reaction Kit and an automated sequencer, ABI 3100 (Applied Biosystems, Carlsbad, CA). Sequences of the primers that were employed for cDNA fragment amplification are given in Additional file 1: Table S1 and sequences of the primers used for genomic sequencing are available upon request. The sequences of the 5’-end regions of the virus genome were determined using 5’/3 RACE Kit 2^nd^ Generation (Roche). The corresponding cDNA fragments were synthesized and amplified using 5’-end sequence-specific and anchor (5’-GACCACGCGTATCGATGTCGACTTTTTTTTTTTTTTTTV-3’) primers according to protocol provided by the manufacturer. Following agarose-gel purification, fragments were subjected to direct sequence analysis as described above. Virus genome sequences were assembled using the Sequencher 4.9 program (Genecodes, Ann Arbor, MI).

### Computer sequence analysis

Derived Cro1 nucleotide and amino acid sequences were analyzed, aligned and compared with vesivirus sequences available from the GenBank database using MacVector (MacVector, Inc., Cary, NC), EMBOSS [34] and Mega5 [33] software packages. The GenBank accession numbers for complete vesivirus genome sequences were: Canine calicivirus (CaCV), AB070225; Vesicular exanthema of swine virus (VESV A48), U76874; San Miguel Sea Lion virus-1 (SMSV1), AF181081; Primate calicivirus 1 (Pan1), AF091736; Walrus calicivirus (WCV), AF321298; Rabbit vesivirus (RaV), AJ866991; Feline calicivirus F9 strain (FCVF9), M86379; Feline calicivirus F4 strain (FCVF4), D31836; Feline calicivirus F65 strain (FCVF65), AF109465; Feline calicivirus Urbana strain (FCVURB), L40021; Steller sea lion vesivirus-v810 (v810), EF193004; Steller sea lion vesivirus-v1415 (v1415), EF195384; Calicivirus isolate Allston 2008/US (ACV8), GQ475302; Calicivirus isolate Geel 2008/Belgium (GCV8), GQ475303; Calicivirus isolate Allston 2009/US (ACV9), GQ475301). The genome sequence of the 780032I strain was submitted to GenBank and assigned accession number JX047864.

A plot of average similarity for each set of the aligned vesivirus sequences was generated using the Plotcon program from the EMBOSS software package ( http://emboss.open-bio.org/rel/rel6/apps/plotcon.html).

Scanning for the protein domains and motifs in the predicted protein sequences were performed using InterProScan ( http://www.ebi.ac.uk/Tools/pfa/iprscan). Signal peptide and subcellular localization motifs predictions were performed using iPSORT ( http://hc.ims.u-tokyo.ac.jp/iPSORT), PSORTII ( http://psort.nibb.ac.jp/form2.html), SOSUIsignal ( http://bp.nuap.nagoya-u.ac.jp/sosui/sosuisignal/sosuisignal_submit.html), SIG-Pred ( http://bmbpcu36.leeds.ac.uk/prot_analysis/Signal.html), Golgi Predictor ( http://ccb.imb.uq.edu.au/golgi/golgi_predictor.shtml), PTS1 predictor ( http://mendel.imp.ac.at/mendeljsp/sat/pts1/PTS1predictor.jsp), Predotar ( http://urgi.versailles.inra.fr/predotar/predotar.html), and SignalIP ( http://www.cbs.dtu.dk/services/SignalP). Prediction of the membrane associated domains was performed using TMpred Server ( http://www.ch.embnet.org/software/TMPRED_form.html).

Bayesian inference of phylogeny was carried out with Mr.Bayes 3.1.2 software [44]. The analysis was performed using Markov chain Monte Carlo sampling under a general time-reversible model of nucleotide substitution with a gamma distribution of rates and a proportion of invariant sites. The search was run for one million generations with tree sampling occurring every 100th generation. Bayesian posterior probabilities and tree topologies were calculated from the consensus of collected tree samples after excluding the first 25% trees as burn-in. The analyses were performed two times for every set of the sequences. The resulting trees were visualized with FigTree1.3.1 ( http://tree.bio.ed.ac.uk/software/figtree/).

### Generation of multiple-cycle growth curve

Vero cell monolayers in six-well plates were infected with the 780032I strain at MOI=0.01, and virus titers were measured at different time points post infection. Briefly, virus stocks were diluted in a supplemented medium (see above), added to cells and allowed to adsorb for one hour at 37 °C. After the incubation, the inocula were removed, the infected cells were washed and fresh growth medium was added to each well. At 0, 8, 16, and 24 hpi, the plates were frozen and stored at −80 °C. All collected samples were freeze-thawed three times, and cell debris was removed by low speed centrifugation. Aliquots of the clarified supernatants were used to determine virus titers at each time point by plaque assay [45].

## Competing interests

The authors declare that they have no competing interests.

## Authors’ contributions

CSJ, KYG and SVS designed the study. CSJ, GIP and SVS performed research and analyzed the data. CSJ, KYG, AWS, SVS wrote the paper. All authors read and approved the final manuscript.

## Supplementary Material

Additional file 1 **Table S1.** PCR primers used in amplification of the Cro1 genome cDNA fragments. Click here for file
